# Plasma microRNA markers of upper limb recovery following human stroke

**DOI:** 10.1038/s41598-018-31020-5

**Published:** 2018-08-22

**Authors:** Matthew A. Edwardson, Xiaogang Zhong, Massimo S. Fiandaca, Howard J. Federoff, Amrita K. Cheema, Alexander W. Dromerick

**Affiliations:** 1 Georgetown University, Department of Neurology, Washington, DC USA; 2 Georgetown University and MedStar National Rehabilitation Hospital, Center for Brain Plasticity and Recovery, Department of Rehabilitation Medicine, Washington, DC USA; 3 Georgetown University, Department of Biostatistics, Bioinformatics, and Biomathematics, Washington, DC USA; 4 University of California Irvine, Department of Neurology, Irvine, CA USA; 5 University of California Irvine, Department of Neurological Surgery, Irvine, CA USA; 6 University of California Irvine, Department of Anatomy & Neurobiology, Irvine, CA USA; 7 UC Irvine Health System, Irvine, CA USA; 8 Georgetown University, Department of Biochemistry, Washington, DC USA; 9 Georgetown University, Department of Oncology, Washington, DC USA; 10 VA Medical Center, Washington, DC USA

**Keywords:** Regeneration and repair in the nervous system, Stroke

## Abstract

Preclinical investigators have implicated several microRNAs as regulators of gene expression promoting neural plasticity following experimental stroke in rodent models. Our goal was to determine whether similar microRNAs might be identifiable in plasma of humans with variable recovery from stroke. Plasma was collected 19 days post-stroke from 27 participants with mild-moderate upper extremity impairment enrolled in the Critical Periods After Stroke Study (CPASS). MicroRNA expression was assessed using TaqMan microRNA assays. Good clinical recovery was defined as ≥6 point change in the Action Research Arm Test (ARAT) score from baseline to 6 months, with 22 subjects showing good and 5 showing poor recovery. When comparing the good versus poor recovery groups, six microRNAs showed significantly decreased expression – miR-371-3p, miR-524, miR-520g, miR-1255A, miR-453, and miR-583, while 3 showed significantly increased expression - miR-941, miR-449b, and miR-581. MiR-371-3p and miR-941 have previously been associated with neural repair mechanisms; none of the significant microRNAs have previously been associated with stroke. The 9 microRNAs converge on pathways associated with axonal guidance, developmental biology, and cancer. We conclude that plasma microRNAs may be informative regarding human neural repair mechanisms during stroke recovery and probably differ from those seen in experimental stroke models.

## Introduction

Ribonucleic acid (RNA) species include microRNAs (miRNAs or miRs), which are small non-coding ~21 residue RNA species^1^. Initially transcribed from nuclear DNA as a primary miRNA (pri-miRNA) transcript, pri-miRNA is then processed within the nucleus to form precursor miRNA (pre-miRNAs) that are transported to the cytoplasm where they are further processed to form the unique miRNA species that interact and influence messenger RNA (mRNA) expression. The human genome encodes over 2000 miRNAs which help regulate the expressed transcripts of roughly half of all genes^2^. MiRNAs function by either degrading mRNA directly (along with a cleavage protein) or through binding to RNA-induced silencing complexes (RISCs) that inhibit/prevent mRNA translation and thereby decrease the synthesis of specific proteins^1^. MiRNAs are quite stable in plasma^3,4^, where they are protected from enzymatic degradation by transport within exosomes^5^ and high density lipoproteins^6^. As intraluminal exosomal cargos, short nucleotide sequences, like miRNAs, are capable of being transported across the blood-brain barrier^7,8^. Dysregulated plasma miRNAs have also been identified in various forms of cancer^9^ and neurological diseases such as Alzheimer’s^10^, multiple sclerosis^11^ and stroke^12–15^.

While many investigators have studied miRNA expression related to the acute phase of stroke (during the 1^st^ 72 hrs) in both animal models^14,16,17^ and humans^12–15^, few have investigated miRNAs during the recovery phase. Vijayan and colleagues recently discovered 4 stroke-related miRNAs (PC-3p-57664, PC-5p-12969, miR-122-5p and miR-211-5p) that are dysregulated not only in human acute stroke serum samples, but also in human post-mortem ischemic brain tissue and acute mouse stroke models^14^. Within 24–48 hrs of a middle cerebral artery occlusion (MCAO) in rodents there is upregulation of brain-specific miR-124a in brain parenchyma^17^ and peripheral blood^18^. Interestingly, a separate study found that miR-124a was downregulated 7 days post-MCAO in the subventricular zone (SVZ), which was thought to promote neural progenitor cell differentiation during neural repair^19^. Other preclinical investigators found that miR-146a becomes upregulated between 0–7 days post-MCAO^19–21^ and may contribute to oligodendrocyte precursor cell differentiation in the SVZ^19^. To our knowledge, there are no prior studies of miRNA expression during the window of maximum spontaneous biological recovery from stroke in humans (~72 hrs to 3 mo post-stroke)^22,23^. This sensitive period of heightened neural plasticity^24^ is characterized by waves of differential gene expression that are associated with axonal sprouting over the first month^25^, and an increase in synaptic density^26^. The differential gene expression during the sensitive period is regulated, at least in part, by miRNAs^27,28^.

The goal of the current exploratory study was to investigate whether miRNAs identified in human plasma collected during the sensitive period show differential expression between patients with clinically significant versus insignificant recovery from stroke. We hypothesize that differentially expressed miRNAs between these two clinical groups may have previously been described in association with stroke and/or neural repair mechanisms and may converge on genes associated with neural plasticity.

## Results

Twenty-two of 27 clinical participants showed good recovery, as determined by at least a 6 point increase in the Action Research Arm Test (ARAT) score from baseline to 6 mo., while the remaining 5 participants displayed poor recovery (ΔARAT <6). Characteristics for the 27 participants in the good and poor recovery groups are described in Table 1. Despite the small number of participants with poor recovery, the two groups were fairly well matched with regard to gender, cardiovascular comorbidities, and time from stroke onset to baseline blood collection (median 19 days for all 27 participants). The poor recovery group was typically older than the good recovery group (median 72 vs. 62.5 respectively) and had lower baseline ARAT scores (median 4 vs. 22 respectively).

**Table 1:** Participant Characteristics.

	Good Recovery (n = 22) ΔARAT ≥ 6	Poor Recovery (n = 5) ΔARAT < 6
Age, median (IQR)	62.5 (52.3–76)	72 (55–73)
Male, n (%)	11 (50%)	2 (40%)
Female, n (%)	11 (50%)	3 (60%)
Race, n (%)		
African American	18 (82%)	5 (100%)
White	3 (14%)	0
Pacific Islander	1 (5%)	0
Cardiovascular Comorbidities, n (%)		
Atrial Fibrillation	1 (5%)	0
Congestive Heart Failure	3 (14%)	0
Hypertension	19 (86%)	4 (80%)
Hyperlipidemia	14 (64%)	2 (40%)
Diabetes	11 (50%)	2 (40%)
Current Smoker	2 (9%)	0
Stroke Subtype, n (%)		
Ischemic Stroke	20 (91%)	5 (100%)
Hemorrhagic Stroke	2 (9%)	0
Days from stroke to baseline assessment, median (IQR)	18 (13.8–19.8)	20 (19–22)
Baseline ARAT (0–57), median (IQR)	22 (5.3–32.8)	4 (3–31)
6 month ARAT (0–57), median (IQR)	49 (37.3–57)	3 (0–35)
ΔARAT, median (IQR)	20 (17–31.3)	−3 (−4–0)

ARAT = Action Research Arm Test; IQR = Interquartile range.

**Figure 1:**
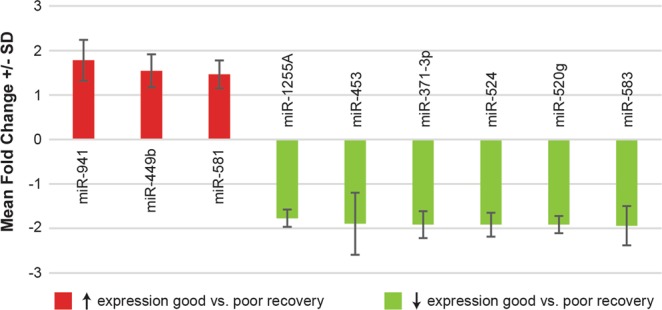
Fold-change for microRNAs with significant differential expression between participants with good (ΔARAT ≥ 6) vs. poor (ΔARAT < 6) recovery of the upper limb. Error bars represent standard deviation.

To investigate differences in miRNA expression between the good and poor recovery groups, we measured plasma miRNA expression using microarray assays. Nine miRNAs were differentially expressed between the good and poor recovery groups (Fig. 1) out of the 754 miRNAs tested. Six miRNAs showed *decreased expression* - miR-371-3p (p = 0.003), miR-524 (p = 0.014), miR-520g (p = 0.015), miR-1255A (p = 0.02), miR-453 (p = 0.037), and miR-583 (p = 0.046); while three showed *increased expression* - miR-941 (p = 0.037), miR-449b (p = 0.043), and miR-581 (p = 0.045). Given the significant imbalance between the good and poor recovery groups, we also performed correlational analysis of the significant miRs, treating ΔARAT as a continuous variable (Table 2). The correlations between ΔARATs for each study participant and miRNA expression levels were in the same direction (positive or negative) as the fold-change for each significant miR. MiR-371-3p and miR-941 showed the strongest correlations (-0.39 and 0.36 respectively). Pathway analyses revealed that the significant miRNAs primarily converge on pathways associated with cancer, axon guidance, and developmental biology (Table 3).

**Table 2:** Fold-change and false discovery rate (FDR) corrected p-values for microRNA expression in participants with good (ΔARAT ≥ 6) vs. poor (ΔARAT < 6) recovery of the upper limb.

	Fold-change	FDR-corrected p-value	Correlation between ΔARAT and miR expression levels
miR-371-3p	1.93 ↓	0.003	−0.39
miR-524	1.93 ↓	0.014	−0.3
miR-520g	1.93 ↓	0.015	−0.34
miR-1255a	1.78 ↓	0.020	−0.17
miR-453	1.91 ↓	0.037	−0.19
miR-941	1.79 ↑	0.037	0.36
miR-449b	1.55 ↑	0.043	0.19
miR-581	1.47 ↑	0.045	0.21
miR-583	1.95 ↓	0.046	−0.23

Correlation between individual ΔARATs and expression levels for each significant miR.

ARAT = Action Research Arm Test.

**Table 3:** Top ten ranked biological pathways identified for the 9 microRNAs differentially expressed between participants with good (ΔARAT ≥ 6) vs. poor (ΔARAT < 6) recovery using 3 different microRNA pathway analysis tools.

Rank	miRSystem	mirPath	Ingenuity Pathway Analysis
1.	Pathways in Cancer	TGF-beta Signaling Pathway	Molecular Mechanisms of Cancer
2.	Axon Guidance	Signaling Pathways Regulating Pluripotency of Stem Cells	Axonal Guidance Signaling
3.	WNT Signaling Pathway	FoxO Signaling Pathway	G-Protein Coupled Receptor Signaling
4.	Axon Guidance	WNT Signaling Pathway	Protein Kinase A Signaling
5.	Developmental Biology	Oocyte Meiosis	Role of Macrophages, Fibroblasts, and Endothelial Cells in Rheumatoid Arthritis
6.	Role of Calcineurin-dependent NFAT Signaling in Lymphocytes	Prostate Cancer	IL-8 Signaling
7.	Prostate Cancer	Hippo Signaling Pathway	Glucocorticoid Receptor Signaling
8.	ERBB1 Downstream Signaling	Central Carbon Metabolism in Cancer	Regulation of the Epithelial-Mesenchymal Transition Pathway
9.	L1CAM Interactions	Proteoglycans in Cancer	Glioblastoma Multiforme Signaling
10.	MAPK Signaling Pathway	Lysine Degradation	Breast Cancer Signaling by Stathmin1

**Figure 2:**
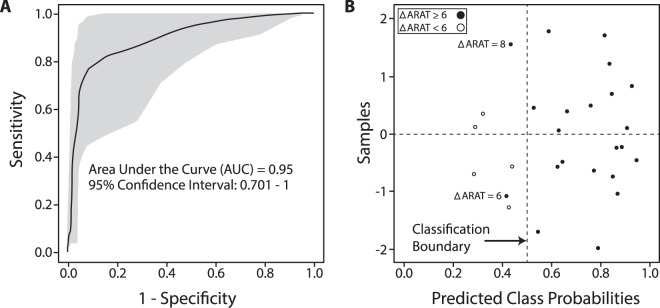
(**A**) Receiver operating characteristic (ROC) curve for good (ΔARAT ≥ 6) versus poor (ΔARAT < 6) recovery using a combination of five miRNAs - miR-581, miR-519b-3p, miR-941, miR-449b, and miR-616. (**B**) Predicted class probabilities for the five miRNA predictive panel, demonstrating 25 correctly classified and 2 misclassified participants. The 2 misclassified participants are labeled by their respective ΔARAT scores.

We performed receiver operating characteristic (ROC) curve analysis to determine whether miRNA biomarkers could accurately predict good versus poor stroke recovery. The five miRNAs with the highest area under the curve (AUC) - miR-581, miR-519b-3p, miR-941, miR-449b, and miR-616 - produced a combined AUC of 0.95 as shown in Fig. 2. Two of these five miRNAs had high AUCs, but were not included in our list of nine differentially expressed miRs in Table 2 due to FDR-corrected p-values > 0.05 (miR-519b, p = 0.0504; miR-616, p = 0.116). The confusion matrix showed that two participants in the good recovery group (ΔARAT ≥ 6) were misclassified into the poor recovery group (ΔARAT < 6). The two misclassified participants had the lowest ΔARAT scores among those in the good recovery group.

Note that only the 5 miRNAs identified in the ROC curve analysis (miR-941, miR-449b, miR-581, 519b-3p, and miR-616) showed expression in > 1/3 of the overall patient cohort. Thus these 5 miRNAs may represent the most promising biomarkers of upper limb recovery.

## Discussion

Although blood-based biomarkers for neurological health and disease are gaining recognition^11,29–33^, there are currently no clinically relevant blood-based biomarkers for neural repair in humans. Such biomarkers would be extremely valuable in identifying the sensitive period of heightened plasticity known to occur after a stroke, and to allow optimal timing of rehabilitation strategies. Human blood-based biomarkers may also provide insights into specific brain repair biology and help drive translational discoveries using preclinical animal models^34^in. This exploratory clinical study was the first step determining whether plasma miRNAs might hold promise as stroke recovery biomarkers, recognizing the limitations of such a reductionistic approach and likely enhancement through future use of multiomic assessments^35,36^. Through a comparison of plasma from stroke recovery participants with good versus poor recovery, however, we identified 9 miRNAs that showed significant differential expression between the groups. None of these miRNAs, to our knowledge, had been previously reported in human stroke or rodent stroke models.

We found evidence in support of further investigations into plasma miRNAs as stroke recovery biomarkers based on a review of the literature, pathway analysis, and predictive (ROC) analysis. Specifically, miR-371-3p has been shown to increase the likelihood that pluripotent stem cells will differentiate into neural progenitors^37^. MiR-941 is the only human-specific miRNA known to be highly expressed in brain tissue^38^. MiRNAs frequently regulate the expression of the host gene with which they are encoded^39,40^. The host gene for miRNA-941, *DNAJC5*, encodes a cysteine-string protein-α (CSPα)^38^, which is expressed in neurons and involved in presynaptic neurotransmitter release^41,42^. Many of the other significant miRNAs are notably dysregulated in various forms of cancer^43–47^, including 2 (miR-520g, miR-524) that affect proliferation of gliomas^45,46^. The association with cancer may not be coincidental, as the molecular machinery for tumor proliferation and regenerative axonal sprouting often overlap^48^. Our pathway analysis, which requires cautious interpretation (see limitations below), also suggests that the miRNAs converge on cancer-related and neural repair pathways. Pathways like axonal guidance and glioma point directly to neural parenchymal involvement, whereas others, such as WNT signaling and pluripotency of stem cells, are less specific to the CNS, but could contribute to neural repair. None of our miRNAs overlapped with the acute stroke-related miRNAs recently found to be shared between humans and rodents^14^. We suspect this was because our blood samples were collected later post-stroke, capturing regenerative as opposed to injury-related changes in gene expression, and because we did not perform global profiling to identify novel miRNAs. Our predictive panel comprised of 5 miRNAs correctly discriminated between good and poor recovery in 25/27 participants, but should be considered preliminary given the small sample size and the numerical imbalance between our stroke recovery groups.

There are a number of limitations to our study. First, we used upper limb recovery of function as a surrogate for neural repair. While we suspect the miRNAs with significant differential expression had most of their effects within the CNS, we cannot exclude that remodeling in peripheral organ systems may have contributed to our findings in blood plasma. Second, most of the participants achieved clinically significant recovery, and thereby provided unbalanced groups for comparison. This imbalance occurred because the study enrolled patients with mild-moderate impairment at baseline, and most such patients go on to achieve significant recovery. We nonetheless felt it was important to use a 6 point change in the ARAT to separate groups, since this is a level of recovery deemed clinically meaningful to patients^49^. There were, however, strong correlations between many of the significant miRs and the ΔARAT, when ΔARAT was treated as a continuous variable, suggesting many of the significant miRNAs would be good discriminators even if a different definition of recovery was used. Third, we did not control for baseline stroke severity or age given the small number of study participants. Fourth, our study lacked non-stroke controls. Fifth, the miRNA pathway analysis is known to suffer from selection bias^50^ and the known canonical biological pathways are over-represented by cancer and to a lesser extent neurobiology. Sixth, the microarray analysis was limited to the known miRNAs found on the qPCR cards. Global miRNA analysis recently discovered novel miRNAs associated with acute stroke^14^, and future investigators may prefer this method to identify novel miRNAs associated with stroke recovery. Finally, we assessed plasma biomarkers only at a single time-point post-stroke. Longitudinal samples, collected at multiple time-points following stroke, are likely to provide the most relevant insights into the evolution of recovery and the role miRNAs might play over time. In spite of these limitations, we are convinced that there is sufficient evidence to pursue more comprehensive investigations of plasma miRNAs in association with recovery from stroke.

Plasma miRNAs hold promise as biomarkers of spontaneous biological recovery following stroke. Future longitudinal studies with appropriate controls will help determine whether the candidate miRNAs discovered in this study might signal a sensitive period of heightened neural plasticity in humans. If such measures can be validated, they may be useful in optimizing the timing of rehabilitation therapy, thereby reducing the burden of stroke disability.

## Materials and Methods

### Participants

The Critical Periods After Stroke Study (CPASS) was performed at the MedStar National Rehabilitation Hospital (Washington, DC)^24^. The study was approved by the MedStar Health Research Institute IRB (approval # 2014-065) and carried out according to their guidelines and regulations; all participants provided written informed consent. Plasma samples were collected from 27 CPASS participants at the time of enrollment. Arm motor function was assessed at baseline and 6 months post-stroke using the Action Research Arm Test^51^ (ARAT). Inclusion/exclusion criteria featured: *inclusion criteria* - ischemic or hemorrhagic stroke, age ≥21, NIH Stroke Scale (NIHSS) arm motor item ≥1, at least a minimal level of preserved function in the hemiparetic arm^24^, Short Blessed Memory Orientation and Concentration Test score ≤8, follows 2 step commands, no prior injury to limb limiting use, and pre-stroke modified Rankin Score <2; *exclusion criteria* - unable to give informed consent, history of prior stroke with persistent hemiparesis or other disabling neurologic condition, hemispatial neglect (asymmetry > 3 on Mesulam Symbol Cancellation Test), NIHSS sensory item score of 2, NIHSS limb ataxia item ≥1, active or prior psychosis or substance abuse, life expectancy <1 year, and received botulinum toxin injection within 6 months.

### Plasma Collection and Storage

Fasting blood samples were collected by venipuncture at the baseline study assessment between 7–9 AM in EDTA-tubes (Cardinal Health, OH, USA). By collecting blood samples near the time of inpatient rehabilitation admission as opposed to the acute hospitalization we hoped to avoid capturing molecular changes related to the initial injury and instead capture changes associated with spontaneous biological recovery. The blood samples were thoroughly mixed, placed on ice, delivered to the Georgetown Lombardi Cancer Center biorepository, and centrifuged at 2600 RPM for 10 min at 20 °C. Plasma was carefully removed via pipette, being careful not to disturb the adjacent buffy coat. Plasma was collected in 750 μL aliquots and frozen at −80 °C until ready for analysis.

### MicroRNA Analysis

Total RNA, including miRNAs and other small RNA molecules, was isolated from 200 μl of plasma and extracted using the Qiagen miRNeasy Serum/Plasma Kit (QIAGEN, Valencia, CA), according to the manufacturer’s instructions. After extraction, the RNA concentration and purity (OD260/280) were measured using the NanoDrop ND-1000 spectrophotometer (Thermo Fischer Scientific, Waltham, MA), and the RNA integrity number (RIN) was determined using an Agilent 2100 Bioanalyzer Instrument (Agilent, Santa Clara, CA, USA). Reverse‐transcription (RT) was carried out using input amounts of 33 nanograms (ng) of total RNA, with Applied Biosystems Megaplex™ RT Primers, Human Pool A and B v3.0, and enzyme kit. This was followed by a subsequent step of pre‐amplification (12 cycles) using Megaplex™ PreAmp Primers, Human Pool A and B v3.0, to enhance assay sensitivity as recommended by the manufacturer (Life Technologies, Carlsbad, CA). Prior to quantitative reverse transcription-polymerase chain reaction (qRT-PCR), complementary DNAs (cDNAs) were loaded onto 384‐well format miRNA assays plates (Taqman Array Human MicroRNA A + B Cards, V3.0, Applied Biosystems, Foster City, CA). Subsequently, qRT‐PCR was performed on a 7900HT Real‐Time PCR System (Applied Biosystems, Foster City, CA).

### Bioinformatics/Statistical Analysis

Good recovery was defined as a change (Δ) in the ARAT score from baseline (median 19 days post-stroke) to 6 months ≥ 6. A change of 6 points was chosen because prior rehabilitation investigators have determined that this is the minimum level of change on the ARAT scale that is clinically meaningful to stroke patients^49^. After data pre-processing, the miRNA expression values were normalized with log transformation, to stabilize the variance, followed by quantile normalization, to make the empirical distribution of intensities similar across samples. Differential expression between patient groups was assessed using independent samples or Wilcoxon-Mann-Whitney U tests. Significance (p) values are reported after adjustment for multiple comparisons, using the false discovery rate (FDR) approach by Benjamini and Hochberg^52^. MiRNAs with differential expression between the two groups, using FDR-corrected p < 0.05, were considered significant. Pearson correlations were determined using the ΔARAT for each individual participant and the expression of each significant miRNA. Analysis was performed using a custom algorithm developed in the ‘R’ programming language. Receiver operating characteristic curve analysis was performed using MetaboAnalyst v4.0 (http://www.metaboanalyst.ca/faces/home.xhtml)^53^.

### Literature Review/Pathway Analysis

We searched PubMed (https://www.ncbi.nlm.nih.gov/pubmed) for miRNAs with significant differential expression previously associated with either stroke and/or neural repair mechanisms within the central nervous system (CNS). We then utilized 3 different miRNA pathway analysis tools (miRSystem v20160513^54^, DIANA mirPath v3.0^55^, and Ingenuity Pathway Analysis (Qiagen, Venlo, NL), to determine whether these preliminary miRNAs show convergence on genes regulating stroke or plasticity-related biological pathways.

## Data Availability

The data discussed in this publication have been deposited in NCBI’s Gene Expression Omnibus^56^ (https://www.ncbi.nlm.nih.gov/geo/query/acc.cgi?acc = GSE114897).
