# Author Correction: Plasma microRNA markers of upper limb recovery following human stroke

**DOI:** 10.1038/s41598-020-58943-2

**Published:** 2020-02-07

**Authors:** Matthew A. Edwardson, Xiaogang Zhong, Massimo S. Fiandaca, Howard J. Federoff, Amrita K. Cheema, Alexander W. Dromerick

**Affiliations:** 1 Georgetown University, Department of Neurology, Washington, DC USA; 2 Georgetown University and MedStar National Rehabilitation Hospital, Center for Brain Plasticity and Recovery, Department of Rehabilitation Medicine, Washington, DC USA; 3 Georgetown University, Department of Biostatistics, Bioinformatics, and Biomathematics, Washington, DC USA; 4 University of California Irvine, Department of Neurology, Irvine, CA USA; 5 University of California Irvine, Department of Neurological Surgery, Irvine, CA USA; 6 University of California Irvine, Department of Anatomy & Neurobiology, Irvine, CA USA; 7 UC Irvine Health System, Irvine, CA USA; 8 Georgetown University, Department of Biochemistry, Washington, DC USA; 9 Georgetown University, Department of Oncology, Washington, DC USA; 10 VA Medical Center, Washington, DC USA

Correction to: *Scientific Reports* 10.1038/s41598-018-31020-5, published online 22 August 2018

The original version of this Article contained a repeated error in which the signs for change in miRNA expression between the good and poor recovery groups were flipped in-text, in Figure 1 and in Table 2.

In the Abstract,

“When comparing the good versus poor recovery groups, six microRNAs showed significantly increased expression – miR-371-3p, miR-524, miR-520g, miR-1255A, miR-453, and miR-583, while 3 showed significantly decreased expression - miR-941, miR-449b, and miR-581.”

now reads:

“When comparing the good versus poor recovery groups, six microRNAs showed significantly decreased expression – miR-371-3p, miR-524, miR-520g, miR-1255A, miR-453, and miR-583, while 3 showed significantly increased expression - miR-941, miR-449b, and miR-581.”

In the Results,

“Six miRNAs showed increased expression - miR-371-3p (p = 0.003), miR-524 (p = 0.014), miR-520g (p = 0.015), miR-1255A (p = 0.02), miR-453 (p = 0.037), and miR-583 (p = 0.046); while three showed decreased expression - miR-941 (p = 0.037), miR-449b (p = 0.043), and miR-581 (p = 0.045).”

now reads:

“Six miRNAs showed decreased expression - miR-371-3p (p = 0.003), miR-524 (p = 0.014), miR-520g (p = 0.015), miR-1255A (p = 0.02), miR-453 (p = 0.037), and miR-583 (p = 0.046); while three showed increased expression - miR-941 (p = 0.037), miR-449b (p = 0.043), and miR-581 (p = 0.045).”

Also, in the Results,

“MiR-371-3p and miR-941 showed the strongest correlations (0.39 and −0.36 respectively).”

now reads:

“MiR-371-3p and miR-941 showed the strongest correlations (−0.39 and 0.36 respectively).”

Additionally, the following statement was previously omitted from the Results section:

“Note that only the 5 miRNAs identified in the ROC curve analysis (miR-941, miR-449b, miR-581, 519b-3p, and miR-616) showed expression in >1/3 of the overall patient cohort. Thus these 5 miRNAs may represent the most promising biomarkers of upper limb recovery.”

These errors have been corrected in the HTML and PDF version of this Article.

